# Bisphenol F exposure induced vascular toxicity through intestinal microbiota imbalance

**DOI:** 10.3389/fmicb.2025.1622488

**Published:** 2025-07-30

**Authors:** Jianlong Yan, Yanbin Pan, Huadong Liu, Jie Yuan, Jie Chen, Yannan Gao, Chaolan Lin, Feng Lin, Rongning Wang, Yaqiong He, Caiping Wang, Cong Xu, Tangzhiming Li, Peng Zhang, Yu Lan, Wenming Shao, Xinli Pang, Da Yin, Xin Sun, Weixiang Luo

**Affiliations:** ^1^Department of Cardiology, Shenzhen Cardiovascular Minimally Invasive Medical Engineering Technology Research and Development Center, Shenzhen People’s Hospital (The Second Clinical Medical College, Jinan University; The First Affiliated Hospital, Southern University of Science and Technology), Shenzhen, Guangdong, China; ^2^Department of Health Management Center, Shenzhen People’s Hospital (The Second Clinical Medical College, Jinan University; The First Affiliated Hospital, Southern University of Science and Technology), Shenzhen, Guangdong, China; ^3^Department of Emergency, The First Affiliated Hospital of Jinan University, Guangzhou, Guangdong, China; ^4^Nursing Department, Shenzhen People’s Hospital (The Second Clinical Medical College, Jinan University; The First Affiliated Hospital, Southern University of Science and Technology), Shenzhen, Guangdong, China

**Keywords:** bisphenol F, faecal microbiota transplantation, gut microbiota, inflammation, short-chain fatty acids, vascular calcification

## Abstract

**Introduction:**

Bisphenol F (BPF), a common substitute for bisphenol A (BPA), has documented toxicity in multiple organs, but its vascular effects remain unclear. This study investigated BPF’s role in vascular calcification (VC) and underlying mechanisms.

**Methods:**

Differences in the intestinal microbiota were analyzed by 16S ribosomal RNA gene sequencing. Metabolites were analyzed using liquid chromatography-mass spectrometry. Faecal microbiota transplantation and antibiotic treatment experiments were performed to evaluate the functions of the intestinal microbiota in VC.

**Results:**

We enrolled consecutively 57 patients. Patients were assigned to a calcification group (30 patients) and a non-calcification group (27 patients) based on the presence or absence of calcification in the thoracic aorta wall. The results showed that patients with vascular calcification (VC) had higher levels of bisphenol F (BPF), bisphenol S (BPS) and bisphenol A (BPA) in the fecal samples than patients without VC. The thoracic aortic calcification score was significantly positively correlated with the BPF (Spearman *r* = 0.4935, *p* < 0.001), BPA (Spearman *r* = 0.2860, *p* < 0.05) and BPS (Spearman *r* = 0.2650, *p* < 0.05). We then explored the effects of BPF exposure on normal and vitamin D3 + nicotine (VDN)-treated rats. BPF exposure induced mild VC in normal rats and aggravated VC in VDN-treated rats. BPF exposure disturbed the gut microbiota and promoted inflammatory responses.

**Conclusion:**

The results here elucidate the mechanism underlying BPF-triggered or BPF-aggravated VC through the gut–vascular axis and provide a theoretical basis for cardiovascular disease risk assessment in humans.

## 1 Introduction

Although bisphenol A (BPA) remains the most extensively studied bisphenol, growing recognition of its health risks has prompted regulatory restrictions and replacement with alternatives in consumer products. BPF has emerged as a prevalent industrial substitute for BPA and widely used in various areas of life and industry, such as construction materials (industrial floors, coatings, and electrical varnishes) (Rochester and Bolden, 2015; Czarny-Krzymińska et al., 2023), consumer products (water pipes, plastics, food packaging, and dental sealants) (Rochester and Bolden, 2015), and paper products (currencies, tickets, and airplane boarding passes) (Liao and Kannan, 2014). However, due to incomplete polymerization during product manufacture as well as the aging and degradation of the product as it is used over time, bisphenols may migrate from the container into food (Kang et al., 2006). In addition, the random discarding and unreasonable recycling of plastic products and thermal paper can cause bisphenols to diffuse into environmental media, such as water, soil, river sediment, atmosphere, and indoor dust (Liao et al., 2012; Yang et al., 2014; Lambré et al., 2023). In daily life, the bisphenols in food, necessities and various environmental media can enter the human body through the digestive tract, skin, or respiratory tract, extensively exposing the human body to bisphenols (Zhang et al., 2018). Currently, BPF can be detected in biological samples from different populations, such as infants (Zheng et al., 2024), pregnant women (Hu et al., 2019), children (Chen et al., 2018), adults (Li et al., 2023), and elderly individuals (Gao et al., 2021). After BPF enters the human body, it can potentially harm multiple human systems.

BPA has been reported to cause a variety of adverse biological effects in the body. For example, zebrafish larvae exposed to BPF displayed disruptions in the regulatory functions of the hypothalamus–pituitary–thyroid axis, with the main manifestation being thyroid endocrine toxicity (Huang et al., 2016). After exposure to an environmentally relevant concentration of BPF (0.0005 mg/L), zebrafish embryos display activated astrocyte- and microglia-mediated neuroinflammation, induced central nervous system cell apoptosis, and inhibited neural development (Yuan et al., 2019). In addition, some studies have shown that exposure to BPF can cause neuronal loss in the zebrafish brain, impairing cognitive ability (Mu et al., 2022a,b). Moreover, exposure to BPF could affect the antioxidant defense system and destroy the metabolic function of the liver (Meng et al., 2019). Intestinal microbial community disorders, oxidative damage and inflammation can be caused by BPF exposure in the zebrafish intestine (Wang et al., 2021). Furthermore, exposing zebrafish to BPF could disrupt the regulatory functions of the hypothalamus–pituitary–gonadal axis and inhibit their pawning capacity, while parental BPF exposure affects the development of the offspring, which mainly manifests as a decreased heart rate, reduced body length, and the inhibition of spontaneous movement (Mu et al., 2022a,b). BPF can also inhibit angiogenesis in zebrafish by increasing oxidative stress (Ji et al., 2022). Moreover, BPF induced bradycardia in zebrafish embryos and potentiated the cardiotoxicity of calcium channel blockers (Arrokhman et al., 2023). Another study showed that cardiotoxicity was induced in mice exposed to BPF for 14 weeks, which mainly manifested as myocardial hypertrophy and cardiac dysfunction (Cheng et al., 2023). Although studies of the toxic effects of BPF have focused on animal experiments, there is growing concern about the potential toxic effects of BPF to humans.

Cardiovascular disease (CVD) is the leading cause of death worldwide (Mahmood et al., 2014). The pathogenesis and development of CVDs are influenced by many factors, including genetics and the environment (Lu et al., 2022). However, after continued in-depth study of the causes and mechanisms of CVDs, it has been found that genetic variations account for only a small portion (<20%) of CVD risk (Ripatti et al., 2010; Ardissino et al., 2011). Therefore, environmental factors play decisive roles in the pathogenesis of CVD, among which exposure to environmental pollution is a critical factor (Münzel et al., 2023). As an environmental pollutant, BPF affects the cardiovascular system, but its effect and mechanism on vascular calcification (VC) are still poorly understood. VC is a common pathological phenomenon in which hydroxyapatite crystals are deposited in the extracellular matrix and between the endarterium and adventitia. VC is the common basis of cardiovascular and cerebrovascular diseases and can lead to arterial stiffness, reduced plasticity, and increased pulse wave velocity, predisposing individuals to CVD events such as heart failure, myocardial infarction, cerebral infarction, and peripheral vascular disease. However, to date, the effect of BPF on vascular toxicity, particularly VC, remains unclear (Dias et al., 2022; Ji et al., 2022; Kang et al., 2023). This underscores a significant knowledge gap in environmental toxicology. Therefore, it is necessary to investigate the effect of BPF on VC.

In this study, we aimed to determine whether there are differences in urine BPF between individuals with VC and those without VC and to investigate the effects of BPF on VC, which provides a new understanding of the effect of BPF on blood vessels and provides a theoretical basis for the risk assessment of BPF-induced cardiovascular disease.

## 2 Materials and methods

### 2.1 Study participants

Fifty-seven consecutive patients with chest pain, including 30 patients with VC and 27 patients without VC, were included in this study. The inclusion criterion was completion of diagnostic thoracic computed tomography imaging. Exclusion criteria were defined as follows: (I) history of gastrointestinal surgery within 12 months, (II) active gastrointestinal disorders (including but not limited to diarrhea, constipation, or bleeding) within 3 months preceding enrollment, (III) comorbid malignancy, autoimmune disease, active infection, or severe renal impairment (serum creatinine >267 μmol/L), (IV) antibiotic, probiotic, or prebiotic use exceeding 3 consecutive days during the 3-month pre-enrollment period, (V) non-compliance with study protocols or withdrawal of informed consent, and (VI) incomplete baseline datasets or missing biological specimens. This observational study was registered under trial NCT04864457. All selected participants provided informed consent, and the research protocol was approved by the Ethics Committee of Shenzhen People’s Hospital. The VC scores were calculated in a manner similar to the calculation of the Agatston calcification score (Agatston et al., 1990); that is, the sum of the calcification scores for the ascending aorta, the aortic arch and the thoracic aorta (from the root of the ascending aorta to the diaphragm) were used as the aortic calcification score (Pedrosa et al., 2019). In addition, basic clinical information of the participants, including age, sex, medical history, etc., was collected.

### 2.2 BPF treatment

The animal experimental protocols complied with the requirements of the Animal Management Committee of Shenzhen People’s Hospital for experimental animal operation and animal welfare. Seven- to eight-week-old male Sprague–Dawley rats were purchased from the Guangdong Medical Experimental Animal Center. The rats were housed in the specific pathogen-free animal room of the Innovation Collaborative Center of Shenzhen People’s Hospital. The rearing conditions were as follows: the animal rooms were alternately light and dark for 12 h each, the relative humidity was 50–70%, and the room temperature was 25 ± 1°C. Each group contained 5 rats housed in two cages (3 and 2 rats per cage, respectively), and they were given free access to food and water. The animals were allowed to adapt to the environment for 1 week, after which the experiment began.

We constructed a VC model according to methods described in previous literature, with slight modifications (Yan et al., 2022). In brief, on the first day, the rats received vitamin D3 (300,000 IU/kg) via intramuscular injection and nicotine (25 mg/kg) via gavage, followed by an additional gavage of nicotine 9 h later. To assess the effect of BPF in normal rats and rats with vitamin D3 + nicotine (VDN)-induced VC, we established four experimental groups, as follows: (i) wild-type rats exposed to olive oil as the normal group (normal group), (ii) rats with VDN-induced VC exposed to olive oil as a VC model (VC group), (iii) wild-type rats exposed to BPF (1 mg/kg body weight/day) (normal + BPF group), and (iv) rats with VDN-induced VC exposed to BPF (1 mg/kg body weight/day) (VC + BPF group). BPF was dissolved in olive oil to prepare a 1 mg/mL solution, which was administered daily at a dose of 1 mg/kg body weight. The exact volume administered to each rat was calculated based on its individual body weight. Following 120 days of BPF exposure, all rats were anesthetized using gas inhalation (3–4% isoflurane with 1–2 L/min oxygen flow during induction, followed by 1.5–2.5% isoflurane at 1 L/min oxygen flow for maintenance) and subsequently euthanized. Rat feces and plasma were collected, and the aortas were fixed in 4% paraformaldehyde, and the remaining tissues were stored at −80°C for future use.

Previous studies have employed varying BPF dosages (0.01–200 mg/kg body weight/day) (Castro et al., 2015; Ullah et al., 2018; Zhao et al., 2018; Ullah et al., 2019; Lee et al., 2022). The lowest observed adverse effect level (LOAEL) for BPF is established at 20 mg/kg, a dose associated with acute hepatorenal dysfunction (Rochester and Bolden, 2015; Zhao et al., 2018). In the present study, we administered 1 mg/kg/day BPF – a dosage substantially below this toxicological threshold (5% of LOAEL). Consequently, adverse effects at this exposure level were significantly milder relative to established toxicity benchmarks.

### 2.3 Fecal microbiota transplantation (FMT)

To determine the changes induced by BPF in the gut microbiota in animals with VC, the feces of the rats in the VC + BPF group (donors) were collected to prepare a fecal bacteria solution. The fecal bacteria solution was prepared according to methods described in previous literature (Staley et al., 2017; Zheng et al., 2021). In brief, fresh feces from donor rats were collected and immediately mixed with sterile PBS (1×) at a ratio of 1:10 (m:v) and vortexed vigorously for 40 s using a benchtop vortex. The mixture was then centrifuged at 1,000 × g for 3 min at 4°C, the collected supernatant was further centrifuged at 6,000 × *g* for 15 min at 4°C after which this supernatant was discarded, and the pellet was resuspended in sterile PBS (1×) to obtain the fresh fecal bacteria solution. The rats in the VC group (recipients) received gavage of the fecal microbial solution once a day based on body weight (a dose of 5 mL/kg) for 14 consecutive days. The group of recipient rats that received the fecal bacteria solution from the rats in the VC + BPF group were named the VC + FMT group.

### 2.4 Depletion of the gut bacteria with the antibiotic cocktail

To study the key role of the gut microbiota in VC, the rats in the VC + BPF group were intragastrically administered antibiotics (ABX) to deplete the gut microbiota, which consisted of 1 mg/mL ampicillin, 1 mg/mL neomycin sulfate, 1 mg/mL metronidazole, 0.5 mg/mL vancomycin hydrochloride, and 1 mg/mL gentamicin dissolved in 0.9% sodium chloride solution (Zhao et al., 2020). The rats were gavaged with Abx at a dosage of 10 μL/g body weight/day once each week, and each gavage was performed continuously for 7 days. Rats in the control group were given the same 0.9% sodium chloride solution without ABX.

### 2.5 Determination of calcification

Samples were fixed in 4% paraformaldehyde, dehydrated, cleared, soaked in paraffin, embedded, sectioned (5 μm), baked, dewaxed, and stained with 2% alizarin red solution (pH = 4.2) for 5 min before being observed under a light microscope (Nikon Eclipse 80i, Tokyo, Japan) and photographed. Image analysis was performed using Image-Pro Plus software (Version 6.0; Media Cybernetics, Rockville, MD, United States).

### 2.6 Biochemical parameters

The serum concentrations of lipopolysaccharide (LPS), diamine oxidase (DAO), interleukin-1β (IL-1β), interleukin-6 (IL-6), and tumor necrosis factor-*α* (TNF-α) were assessed using enzyme-linked immunosorbent assay (ELISA) kits obtained from Jianglai Biotech, Shanghai, China. All biochemical parameters were analyzed following the manufacturers’ protocols.

### 2.7 DNA sequencing of the microbiota

Total DNA extraction was completed by the cetyltrimethylammonium bromide (CTAB) method (Yan et al., 2022). The concentration and purity of the extracted DNA were determined via 1% agarose gels. PCR amplification was carried out for the V3-V4 variable regions in the 16S rRNA genes of bacteria using the primers 341F (5’-CCTAYGGGRBGCASCAG-3′) and 806R (5′- GGACTACNNGGGTATCTAAT-3′). The amplification procedure was as follows: predenaturation at 98°C for 1 min; 30 cycles of denaturation at 98°C for 10 s followed by annealing at 50°C for 30 s and extension at 72°C for 30 s; and a final extension at 72°C for 5 min (PCR instrument: T100PCR, Bio-Rad, United States). Then, 2% agarose gel electrophoresis was performed to detect the PCR products, and a Qiagen Gel Extraction Kit (Qiagen, Germany) was utilized for purification. Finally, a TruSeq^®^ DNA PCR-Free Sample Preparation Kit (Illumina, United States) was used to construct a library, and sequencing was conducted on an Illumina NovaSeq 6,000 PE250 (Novogene, Tianjin, China).

### 2.8 Bioinformatic analysis

In this study, the analytical processes used were consistent with those described in our previously publication (Yan et al., 2022). First, the raw sequence data were processed using Quantitative Insights into Microbial Ecology 2 (QIIME2, version 2020.2) software. This included application of the dada2 plugin QIIME2 to perform quality control, truncation, denoising, connection, and chimera removal to generate the final representative sequence list. Subsequently, using the feature classifier plugin QIIME2, the representative amplicon sequence variants (ASVs) were matched to the Silva database to identify the corresponding taxa and determine their abundance. To identify potential biomarkers in the microbial communities, we used the linear discriminant analysis effect size (LEfSe) method for difference analysis. In addition, we used the observed ASVs and the Shannon index to measure the microbial diversity (*α* diversity) in the samples. Finally, the differences between different community structures were explored by principal coordinate analysis (PCoA) and permutation multivariate analysis of variance (PERMANOVA) (*p < 0.05, **p < 0.01, ***p < 0.001, ****p < 0.0001).

### 2.9 Plasma sample pretreatment

First, 100 μL of serum was aliquoted into individual 1.5 mL polypropylene extraction tubes. Methanol was then added at a 1:4 (v/v) ratio and the mixture was vortexed thoroughly. Following centrifugation at 20,000 rpm for 20 min (4°C), the supernatant was carefully collected. Subsequently, 300 μL of ethyl acetate and 10 μL of 0.5% formic acid were added to the supernatant. The mixture was vortex-mixed for 30 s and centrifuged at 20,000 rpm for 3 min (4°C) to separate the ethyl acetate layer. This extraction procedure was repeated twice more with fresh ethyl acetate. All ethyl acetate fractions were pooled for derivatization (Zhu et al., 2016; Zheng et al., 2018).

### 2.10 Fecal sample pretreatment for extracting SCFAs

A stool sample (150–200 mg) was placed in a polypropylene extraction tube, and 1.0 mL of acetonitrile (CAN) was added. Next, the mixture was vortexed for 3 min. Afterward, centrifugation was performed at 20,000 rpm for 3 min at 4°C, and the supernatant was carefully collected. This centrifugation step was repeated three times, and the supernatant was carefully collected each time and retained for subsequent derivatization experiments (Yuan et al., 2018).

### 2.11 Fecal sample pretreatment for extracting bisphenols

Accurately weighed fecal samples (2–3 g) were transferred into conical flasks. Then, 20 mL of 50% (v/v) methanol was added for extraction, followed by incubation for 2.0 h at room temperature. After extraction, 10 mL of supernatant was transferred to a pre-conditioned Oasis HLB solid-phase extraction (SPE) cartridge (pre-activated sequentially with 5 mL methanol and 5 mL pure water). The initial eluate was discarded, and the cartridge was washed successively with 5 mL pure water and 8 mL methanol–water (1:1, v/v). Target analytes were eluted with 6 mL pure methanol at a controlled flow rate of 1 mL/min. The eluate was concentrated to near-dryness under a gentle nitrogen stream. The residue was reconstituted in 1 mL methanol–water (1:1, v/v) and filtered through a 0.22 μm membrane prior to instrumental analysis.

### 2.12 Derivatization

A total of 30 μL (20 μmol/mL) of triethylamine (TEA) and 15 μL (20 μmol/mL) of 2-chloro-1-methylpyridinium iodide (CMPI) were added to the above sample, followed by homogeneous mixing using a vortex mixer. The mixture was incubated at 40°C for 5 min. Next, 30 μL (20 μmol/mL) N, N-Dimethylethylenediamine (DMED) was added, and incubation continued at 40°C for 60 min. After that, the sample was dried under a nitrogen flow, and 100 μL of Acetonitrile (ACN) can was added (Zhu et al., 2016; Yuan et al., 2018; Zheng et al., 2018).

### 2.13 Liquid chromatography–mass spectrometry analysis for SCFAs

This study used a method similar to that described in our previously published study (Yan et al., 2022). In brief, the LC conditions were as follows: the column used was an ACQUITY UPLC HSS T3 column (100 Å, 1.8 μm, 2.1 mm × 100 mm) (Waters, Milford, MA, United States); and mobile phase A consisted of water (containing 0.1% formic acid) and mobile phase B consisted of ACN (containing 0.1% formic acid). Gradient elution was performed as follows: 0 ~ 5 min, 2–10% B; 5 ~ 13 min, 10% ~ 100% B; 13 ~ 15 min, 100% B; 15 ~ 16 min, 100% ~ 2% B; and 16 ~ 20 min, 2% B. The flow rate was 0.2 mL/min, the temperature of the sample chamber was 10°C, the column temperature was 25°C, and the injection volume was 2 μL. MS conditions: The Q Exactive Focus system (Thermo Fisher Scientific, Santa Clara, CA, United States) was operated in positive ion mode with electrospray ionization: Orbitrap; ion source: HESI; scan type: full MS; heater temperature, 350°C; capillary temperature, 320°C; sheath gas flow, 40 arbitrary units; auxiliary gas flow, 10 arbitrary units; spray voltage, 3.8 kV; capillary voltage, 35 V; S-lens RF level, 50%; maximum injection time, 100 ms; scan range: m/z 70–1,050; and resolution: 70,000 FWHM.

### 2.14 Liquid chromatography–mass spectrometry analysis for bisphenols

Liquid chromatography conditions: The chromatographic column was an Agilent Eclipseplus C18 (100 Å, 1.8 μm, 2.1 mm × 100 mm), column temperature: 40°C; flow rate: 0.3 mL/min; injection volume: 2 μL; mobile phase: water (A) – methanol (B); gradient elution: 0–0.5 min, 95% A; 0.5–2.5 min, 95–40% A; 2.5–6.0 min, 40–5% A; 6.0–7.5 min, 5% A; 7.5–7.51 min, 95 A%; 7.51–12 min, 95 A%. Mass spectrometry conditions: The analysis was performed using an Agilent 6465B Triple Quadrupole LC–MS system (Agilent Technologies, Santa Clara, CA, United States); Ionization mode: electrospray ionization (ESI-); Scan mode: multiple reaction monitoring mode; Dryer temperature: 350°C; Dryer flow rate: 7 L/min; Nebulizer pressure: 30 psi; Sheath gas temperature: 300°C; Sheath gas flow rate: 11 L/min; Capillary voltage: 2000 V.

### 2.15 Statistical analysis

Statistical analyses were performed using EmpowerStats^1^, R^2^, and GraphPad Prism 8.0. Data are expressed as mean ± standard deviation (SD) for normally distributed variables (analyzed by two-tailed Student’s *t*-test after confirming equal variance via *F*-test) or median with interquartile range (P25, P75) for non-normally distributed variables (analyzed by Kruskal–Wallis rank sum test). Categorical variables are presented as frequencies/percentages and compared using chi-square tests. Correlation analyses were conducted using Spearman’s rho non-parametric correlation. PERMANOVA was performed using OmicShare tools for data^3^. A two-sided *p* < 0.05 defined statistical significance.

## 3 Results

### 3.1 Characteristics of the participants

A total of 57 participants were consecutively enrolled in this study. The results showed that compared with those in the Non-VC group, the participants in the VC group were older, had a lower eGFR, and had higher rates of smoking (*p* < 0.05). There were no significant differences in sex, BMI, total cholesterol, triglyceride, HDL-C, LDL-C, uric acid, serum calcium, serum phosphorus, creatinine, drinking, hypertension, diabetes mellitus (*p* > 0.05). The basic demographic and clinical characteristics of all study subjects are shown in Table 1.

**Table 1:** Characteristics of the participants at baseline.

Characteristics	Non-VC (*n* = 27)	VC (*n* = 30)	*P*-value
Score	0.0 (0.0–0.0)	291.5 (149.1–768.0)	<0.001
Age, (years)	54.0 ± 7.0	60.9 ± 7.3	<0.001
Male, *n* (%)	15 (55.6)	16 (53.3)	0.866
BMI, kg/m^2^	25.4 ± 3.2	24.7 ± 3.0	0.401
Total cholesterol, mmol/L	5.45 ± 1.05	5.33 ± 1.22	0.694
Triglyceride, mmol/L	1.26 (1.14–1.74)	1.31 (1.06–1.98)	0.765
HDL-C, mmol/L	1.48 ± 0.39	1.44 ± 0.39	0.708
LDL-C, mmol/L	3.00 ± 0.80	2.93 ± 0.94	0.761
Uric acid, mmol/L	406.0 ± 102.1	417.4 ± 92.1	0.660
Serum calcium, mmol/L	2.42 ± 0.08	2.39 ± 0.09	0.221
Serum phosphorus, mmol/L	1.14 ± 0.14	1.12 ± 0.17	0.681
Creatinine, μmol/L	69.0 ± 15.3	73.5 ± 13.1	0.236
eGFR (mL/min/1.73 m^2^)	97.0 ± 12.2	87.5 ± 13.4	0.008
Smoking, *n* (%)	4 (14.8)	12 (40.0)	0.035
Drinking, *n* (%)	6 (22.2)	7 (23.3)	0.920
Hypertension, *n* (%)	14 (51.9)	16 (53.3)	0.911
Diabetes mellitus, *n* (%)	6 (22.2)	6 (20.0)	0.837
Bisphenol S, (μg/g)	0.008 (0.003–0.013)	0.014 (0.007–0.024)	0.003
Bisphenol F, (μg/g)	0.012 (0.007–0.020)	0.027 (0.015–0.040)	<0.001
Bisphenol A, (μg/g)	0.019 (0.014–0.026)	0.028 (0.018–0.047)	0.006

Data are presented as mean ± standard deviation (SD), median (interquartile range [IQR]) or *n* (%). *P*-value < 0.05 was considered statistically significant. BMI, Body mass index; EGFR, estimated glomerular filtration rate; HDL-C, high density lipoprotein cholesterol; LDL-C, low density lipoprotein cholesterol.

### 3.2 Fecal bisphenols in patients with VC

**Figure 1:**
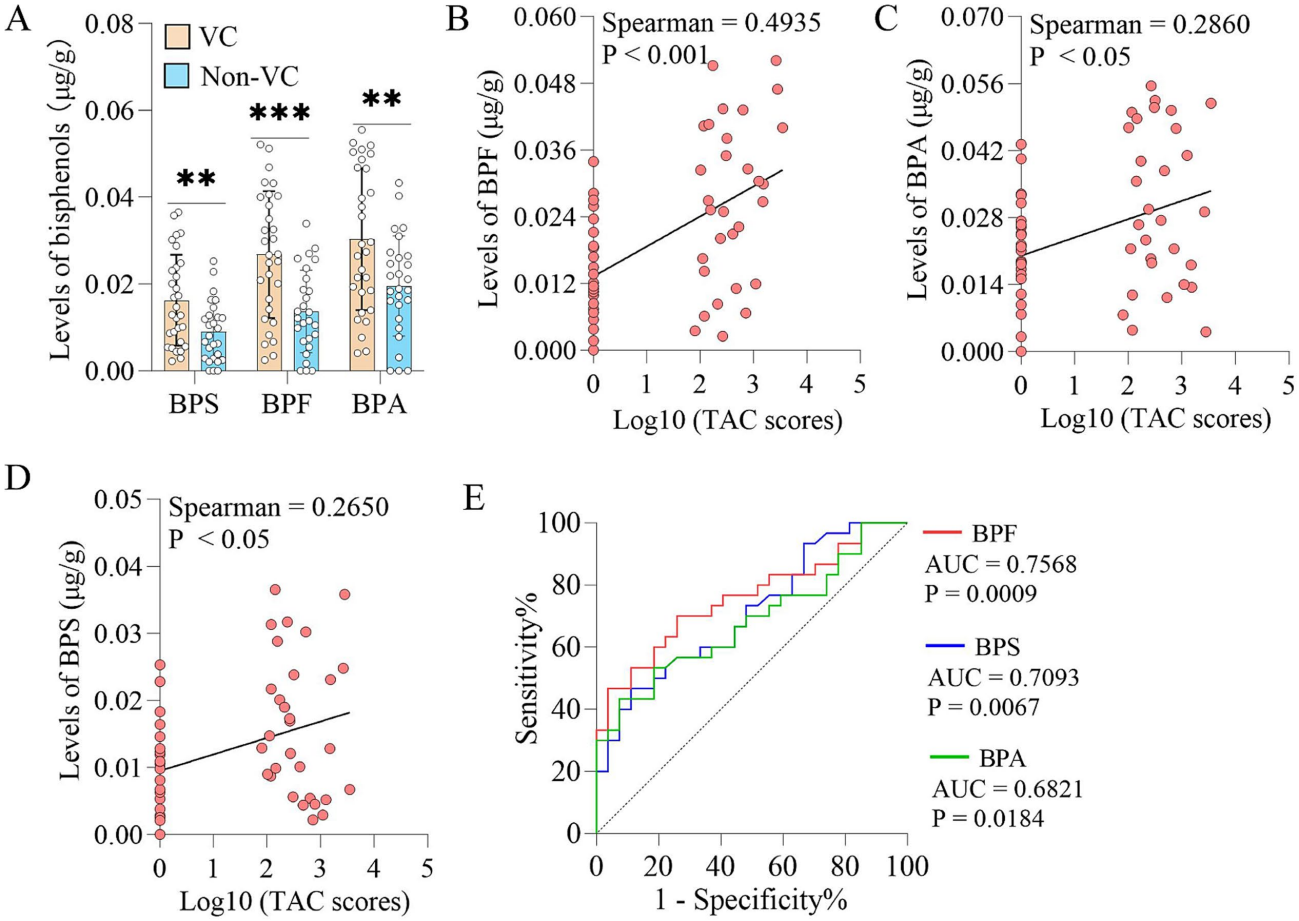
Fecal bisphenols in patients with VC. **(A)** Comparison of the abundances of bisphenols in fecal samples from patients with and without VC. **(B–D)** Spearman’s correlation analyses of BPF, BPA and BPS levels in fecal samples with TAC scores, respectively. **(E)** Predictive ability of BPF, BPA and BPS levels in fecal samples for vascular calcification, respectively. Continuous and non-normal data were described as median (interquartile range). The Mann–Whitney *U* test was used to analyze the differences of two groups. **p < 0.01, ***p < 0.001. BPA, Bisphenol A; BPF, Bisphenol F; BPS, Bisphenol S; TAC, Thoracic aortic calcification; VC, Vascular calcification.

Patients with VC had significantly higher levels of BPS, BPF and BPA in the fecal samples than Non-VC patients (Figure 1A). To further reveal the relationship between bisphenols exposure and VC severity, we conducted a correlation analysis between the levels of bisphenols and the VC score. The results indicated that the VC scores were positively correlated with the levels of BPF (Spearman *r* = 0.4935, *p* < 0.001) (Figure 1B), BPA (Spearman *r* = 0.2860, *p* < 0.05) (Figure 1C) and BPS (Spearman *r* = 0.2650, *p* < 0.05) (Figure 1D). In addition, receiver operating characteristic (ROC) curves were plotted to calculate the area under the curve (AUC) to assess the predictive role of bisphenols for VC. It was discovered that the largest AUC was generated by BPF, followed by BPS, and BPA had the lowest AUC (Figure 1E).

### 3.3 Association between fecal bisphenols and gut microbiota in patients with VC

**Figure 2:**
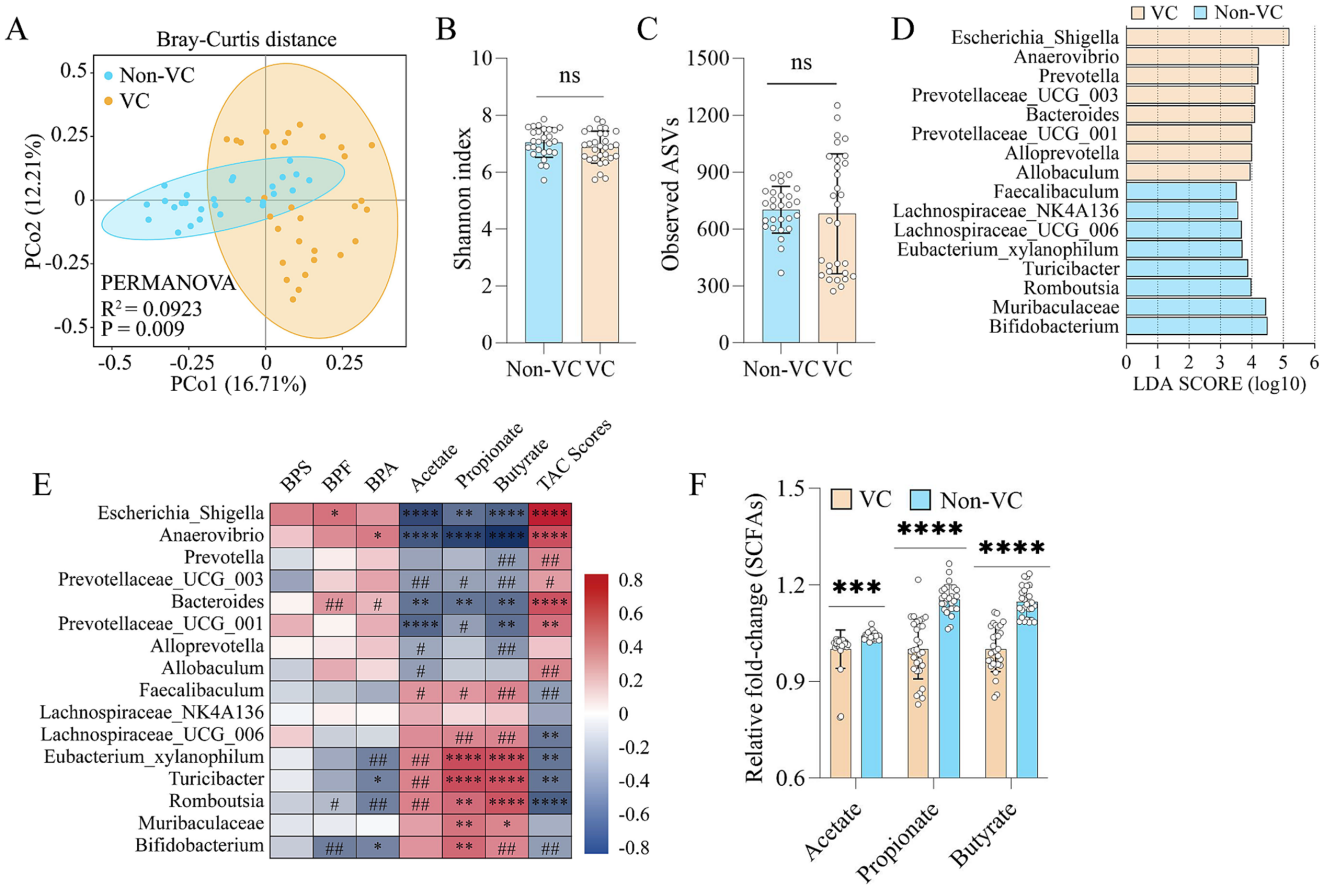
Association between fecal bisphenols and gut microbiota in patients with VC. **(A)** PCoA plot showing changes in the β-diversity of the gut microbiota in patients with and without VC. **(B)** α-diversity of the gut microbiota (Shannon index). **(C)** α-diversity of the gut microbiota (Observed ASVs). **(D)** LEfSe (linear discriminant analysis (LDA) coupled with exact size measurements) for the analysis of differences between the gut microbiota of VC group and Non-VC group (LDA > 3.5). **(E)** Spearman’s correlation analysis of the relationship of the intestinal microbiota with BPF, BPA, BPS, acetate, propionate, butyrate and TAC scores. Negative and positive correlations are denoted in blue and red, respectively. The adjusted *p*-value was calculated with the Benjamini–Hochberg false discovery rate (FDR) method to correct the multiple comparisons and Spearman’s correlations. **(F)** Comparison of the differences in fecal acetate, propionate, and butyrate levels, respectively. Continuous data were described as mean ± standard deviation or median (interquartile range) as appropriate. The *t*-test for normally distributed data and with the Mann–Whitney *U* test for non-normally distributed data. ^#^*p* < 0.25, ^##^*p* < 0.1, *p < 0.05, **p < 0.01, ***p < 0.001, ****p < 0.0001, ns > 0.05.

To reveal the structure and abundance of the intestinal microbiota in vascular calcification patients with higher bisphenols in the fecal samples, we used 16S rRNA gene sequencing to analyze the structure and abundance of the gut microbiota. Principal coordinate analysis (PCoA) at the bacterial phylum level indicated that there were structural differences in the intestinal microbiota between the two groups (*R*^2^ = 0.0923, *p* = 0.009) (Figure 2A), despite non-significant changes in *α*-diversity (Figures 2B,C). Furthermore, linear discriminant analysis (LDA) effect size (LEfSe) indicated that VC were enriched in *Escherichia_Shigella*, *Anaerovibrio*, *Prevotella*, *Bacteroides*, etc. (LDA > 3.5) (Figure 2D). Furthermore, spearman correlation analysis demonstrated that the abundances of *Escherichia_Shigella*, *Anaerovibrio*, *Bacteroides*, etc., were variably positively correlated with BPS, BPF, BPA and TAC scores in the VC group and negatively correlated with acetate, propionate and butyrate levels in feces. Additionally, spearman correlation analysis indicated that the abundances of *Bifidobacterium*, *Romboutsia*, *Eubacterium_xylanophilum*, *Turicibacter*, etc., were positively correlated with the levels of acetate, propionate and butyrate and negatively correlated with BPS, BPF, BPA and TAC scores to different degrees in the Non-VC group (Figure 2E). Further analysis found that patients with VC had significantly lower levels of acetate, propionate and butyrate in the fecal samples than Non-VC patients (Figure 2F).

### 3.4 BPF exposure induced mild VC in normal rats and aggravated VC in VDN-treated rats

**Figure 3:**
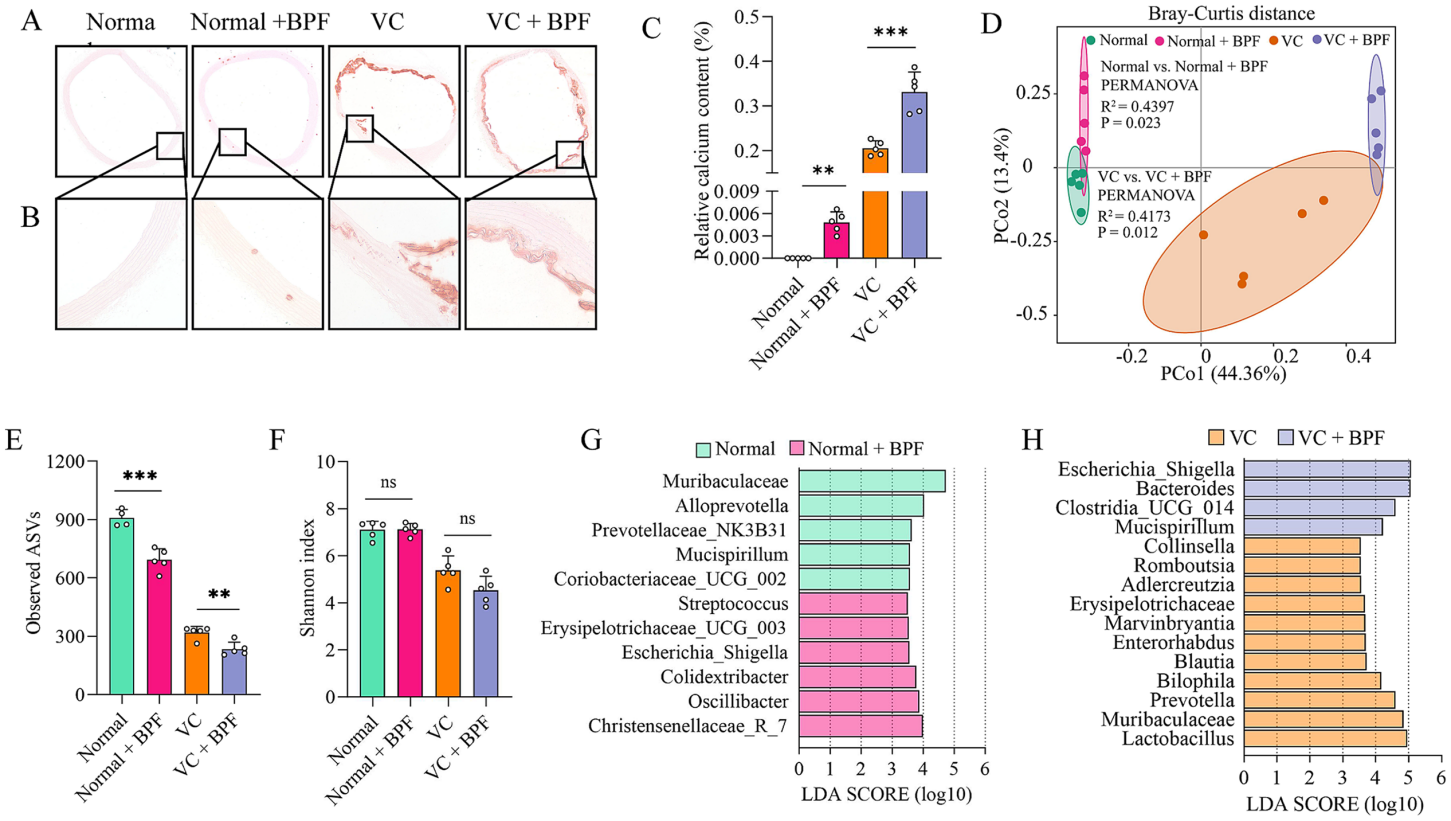
BPF exposure promoted VC and disturbed the gut microbiota. **(A)** Alizarin red staining of ascending aorta calcification (original magnification, 40×) (*n* = 5). **(B)** Alizarin red staining of ascending aorta calcification (original magnification, 200×) (*n* = 5). **(C)** Relative quantification of calcium content for the vessel sections of the ascending aorta (*n* = 5). **(D)** PCoA plot showing changes in the β-diversity of the gut microbiota after BPF treatment (*n* = 5). **(E)** α-diversity of the gut microbiota (Observed ASVs) (*n* = 4 ~ 5). **(F)** α-diversity of the gut microbiota (Shannon index) (*n* = 5). **(G)** LEfSe [linear discriminant analysis (LDA) coupled with exact size measurements] for the analysis of differences between the gut microbiota of the normal group and the normal + BPF group (LDA > 3.5) (*n* = 5). **(H)** LEfSe analysis of the differences between the gut microbiota of the VC group and the VC + BPF group (LDA > 3.5) (*n* = 5). The *t*-test was used to analyze the differences of two groups. ns > 0.05, **p < 0.01, ***p < 0.001.

Based on the ROC curves results, we selected BPF as the study subject. To elucidate the effect of BPF on VC, we exposed rats to BPF for 120 days, after which pathological changes in the ascending aorta were detected. Alizarin red staining revealed that the ascending aortas of the rats in the normal group had no calcification (Figures 3A,B). Compared with those in the normal group, the ascending aortas of the rats in the normal + BPF group had slight calcification (Figures 3A,B). Compared with those in the VC group, the ascending aortas of the rats in the VC + BPF group had more severe calcification (Figures 3A,B). In addition, relative quantification of the calcium salt showed that compared with those in the normal and VC groups, the calcium contents in the ascending aortas of the normal + BPF and VC + BPF groups were significantly increased (Figure 3C). These results indicate that BPF exposure induces mild calcification of the ascending aorta in normal rats and can aggravate VDN-induced ascending aorta calcification.

### 3.5 BPF exposure disturbed the gut microbiota

The gut microbiota is a “microbial organ” (Byndloss and Bäumler, 2018) and can be regulated by a variety of exogenous substances. To clarify the effect of BPF on the gut microbiota, we used 16S rRNA gene sequencing to analyze the structure and abundance of the gut microbiota. After 120 days of BPF treatment, the gut microbiota of the rats were significantly disrupted. PCoA at the phylum level revealed differences in the structure of the gut microbiota after BPF treatment (*R*^2^ = 0.4173, *p* = 0.012) (Figure 3D) and that BPF treatment reduced the *α* diversity of the gut microbiota (observed ASVs) (Figure 3E); however, no significant difference was observed in the Shannon index (Figure 3F). Furthermore, LEfSe revealed that both normal rats and VDN-treated rats exposed to BPF exhibited enrichment of the genus Escherichia_Shigella (LDA > 3.5), while rats not exposed to BPF did not show enrichment of this genus (Figures 3G,H).

### 3.6 BPF exposure promoted systemic inflammatory responses

**Figure 4:**
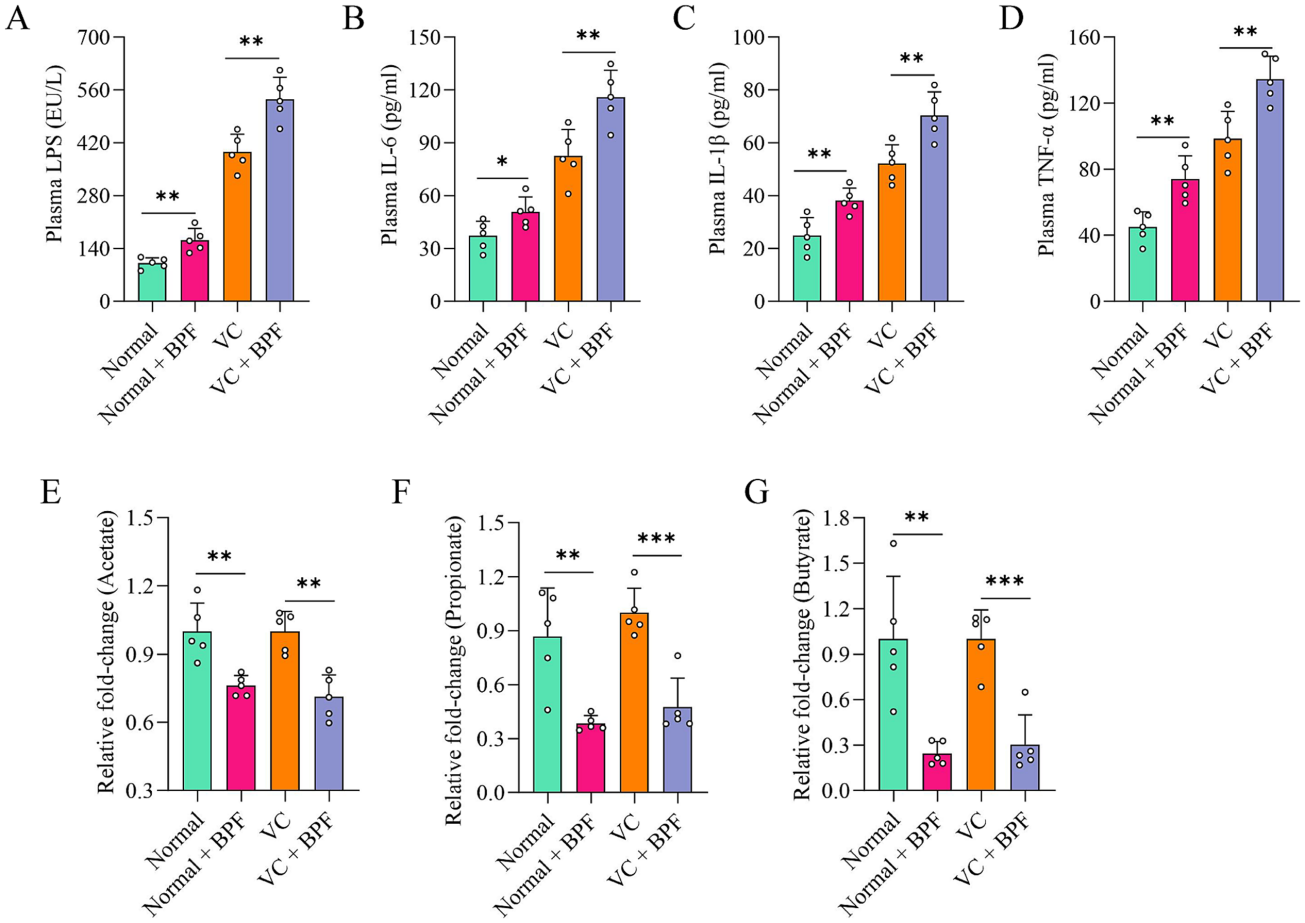
BPF exposure promoted systemic inflammatory responses. **(A)** Comparison of the differences in plasma LPS levels (*n* = 5). **(B–D)** Comparison of the differences in plasma levels of IL-6, IL-1β and TNF-α, respectively (*n* = 5). **(E–G)** Comparison of the differences in fecal acetate, propionate, and butyrate levels, respectively (*n* = 5). The *t*-test for normally distributed data and with the Mann–Whitney *U* test for non-normally distributed data. *p < 0.05, **p < 0.01, ***p < 0.001.

Escherichia_Shigella is one of the main sources of the intestinal endotoxin LPS (Wang et al., 2022). Therefore, we further analyzed the LPS level in plasma and found that they were significantly higher in the normal + BPF group and the VC + BPF group than in the normal group and VC group (Figure 4A). In addition, we measured the plasma levels of biological factors associated with inflammation to assess the effect of BPF on systemic inflammation. Compared with those in the normal group and VC group, the plasma concentrations of IL-6, IL-1β, and TNF-*α* in the normal + BPF group and the VC + BPF group were significantly increased (Figures 4B–D). LPS and short-chain fatty acids (SCFAs) are two types of metabolites derived from the gut microbiota that reflect the status of the intestinal system and the functional status of the gut microbiota (Zhang X. Y. et al., 2020; Zhang X. et al., 2020). We detected the main SCFAs in feces, and the results showed that, compared with the normal group and VC group, the normal + BPF group and the VC + BPF group had decreases in the contents of acetic acid, propanoic acid, and butyric acid to varying extents (Figures 4E–G). Taken together, these results reveal that BPF exposure causes drastic changes in the gut microbiota and promotes inflammation in the body.

### 3.7 BPF promotes VC through the gut microbiota

**Figure 5:**
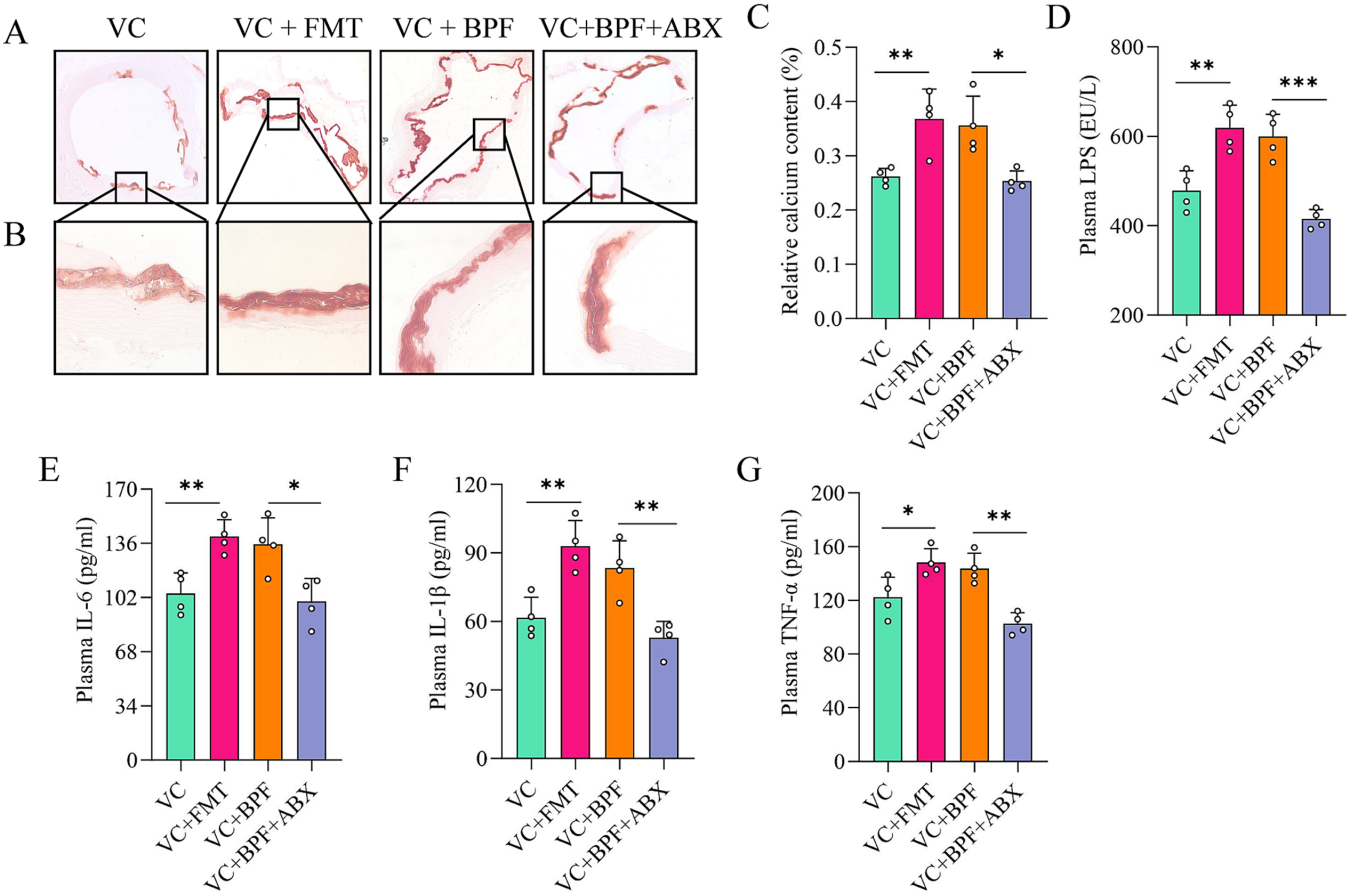
BPF promotes VC through the gut microbiota. **(A)** Alizarin red staining of ascending aorta calcification (original magnification, 40×) (*n* = 4). **(B)** Alizarin red staining of ascending aorta calcification (original magnification, 200×) (*n* = 4). **(C)** Relative quantification of calcium content for the vessel sections of the ascending aorta (*n* = 4). **(D)** Comparison of the differences in plasma LPS levels (*n* = 4). **(E–G)** Comparison of the differences in plasma levels of IL-6, IL-1β and TNF-α, respectively (*n* = 4). The *t*-test was used to analyze the differences of two groups. *p < 0.05, **p < 0.01, ***p < 0.001.

To study the key role of the gut microbiota in promoting VC, we transplanted the fecal microbiota from the rats in the VC + BPF group to the rats in the VC group (named the VC + FMT group). After FMT, VC in the VC + FMT group was exacerbated (Figures 5A–C). In contrast, the rats in the VC + BPF group received antibiotic cocktail (ABX) treatment (named the VC + BPF + ABX group). Compared with that in the VC + BPF group, VC in the VC + BPF + ABX group was significantly reduced (Figures 5A–C). We further analyzed the factors associated with inflammation in plasma, and the results showed that compared with those in the VC group, the plasma concentrations of LPS, IL-6, IL-1β, and TNF-α in the rats in the VC + FMT group increased to different degrees (Figures 5D–G), while the concentrations of LPS, IL-6, IL-1β and TNF-α in the VC + BPF + ABX group decreased to different degrees when compared with that in the VC + BPF group (Figures 5D–G). These results indicate that BPF promotes VC through the gut microbiota.

## 4 Discussion

Bisphenols, a well-known class of endocrine-disrupting chemicals, are widely used in various fields of industry and life. Humans are frequently exposed to bisphenols in daily life, which continuously poses an enormous threat to their health. To our knowledge, this study is the first to report the presence of different concentrations of Bisphenols (BPF, BPF and BPA) in the feces of patients with VC. BPF exposure induced mild VC in normal rats and aggravated VC of VDN-treated rats. The mechanisms may involve BPF exposure-induced perturbation of the gut microbiota and promotion of inflammatory responses.

To our knowledge, only one prior study has reported bisphenol levels in human feces. That study found bisphenols undetectable in some samples. In detectable samples, concentrations ranged as follows: BPA from 47.9 to >500 ng/g; BPF between 28.8 and 400.1 ng/g; and BPS between 43.6 and 83.8 ng/g (Moscoso-Ruiz et al., 2024). Consistent with our findings, BPA showed the highest concentrations, followed by BPF, with BPS being the lowest. However, the bisphenol levels detected in our study were significantly lower than those reported in that research. This discrepancy may stem from differences in the study populations and their environmental exposures (Kang et al., 2006; Liao et al., 2012; Liao and Kannan, 2014; Rochester and Bolden, 2015).

In addition to genetic factors, environmental factors are important in triggering or aggravating CVDs (Lin et al., 2019) that also affect the composition and diversity of gut microbes (Tan et al., 2022). This study revealed that the intestines of rats exposed to BPF were enriched in the genera Escherichia and Shigella, which are associated with the endotoxin LPS and can cause the body to develop a significant inflammatory response. The intestines of BPF-exposed rats were enriched in the gut microbiota associated with the production of SCFAs, such as Alloprevotella, Muribaculaceae, Lactobacillus, Prevotella, Bilophila, Blautia, and Romboutsia; the absence of these symbiotic bacteria is unfavorable for the production of SCFAs (Koh et al., 2016). Our metabolomics data also demonstrated that the SCFA contents in the intestines of rats exposed to BPF were significantly reduced. LPS and SCFAs are two types of important metabolites derived from the gut microbiota that reflect the intestinal system status and the functional status of the gut microbiota (Zhang X. Y. et al., 2020; Zhang X. et al., 2020). Therefore, our study indicated that BPF-related gut microbiota dysbiosis leads to the excessive release of LPS and a deficiency in SCFAs, which may be an important mechanism leading to VC.

To better explain the role of BPF-associated gut microbiota dysbiosis in VC, we transplanted fecal bacteria from BPF-exposed rats to rats with VC and found that FMT significantly aggravated VC and increased the production of inflammatory cytokines. In contrast, Abx treatment depleted the gut microbiota and significantly reduced VC and the production of inflammatory cytokines. Therefore, our study indicated that the exacerbation of VC was dependent on BPF-related gut microbiota dysbiosis, especially the increased abundance of pathogenic Escherichia and Shigella. Escherichia and Shigella, the main members of Proteobacteria, are the main sources of gut-derived LPS (Wang et al., 2022). LPS enters the systemic circulation and activates the Toll-like receptor 4 (TLR4) signaling pathway and TNF-α, IL-1β, and IL-6. The transcription of inflammatory cytokines amplifies the inflammatory response, triggering chronic inflammation in the body (Yan et al., 2023). The development of VC is closely related to the activation of chronic, non-specific inflammation in the body. The results of this study showed that the levels of proinflammatory cytokines, such as LPS, IL-6, IL-1β, and TNF-α, in the systemic circulation of rats exposed to BPF were significantly increased. Consistent with this, BPF exposure disrupted the zebrafish gut microbiota and promoted the expression of the inflammatory cytokines IL-1β and TNF-α (Wang et al., 2021). Inflammatory mediators can activate the inflammation-related transcription factor NF-kB pathway to promote the expression of bone morphogenetic protein-2 (BMP-2), Runt-related transcription Factor 2 (RUNX2) and alkaline phosphatase (ALP) (Panizo et al., 2009; Deuell et al., 2012; Zhang X. Y. et al., 2020; Zhang X. et al., 2020), thereby promoting VC. In summary, exposure to BPF can lead to gut microbiota dysbiosis and the excessive production of LPS, which triggers chronic inflammation in the body and eventually induces or aggravates VC.

The SCFAs are metabolites produced by the gut microbiota that regulate gut microbiota homeostasis (Yan et al., 2022). Our previous study showed that exogenous supplementation with SCFAs (such as propionate) could improve gut microbiota homeostasis (Yan et al., 2022). Studies have shown that when intestinal SCFAs are lacking or insufficient, the pH increases, which is unfavorable for the growth of beneficial bacteria and instead promotes the growth of harmful bacteria, leading to gut microbiota dysbiosis and promoting the development and progression of gut microbiota-related diseases (Wang and Zhao, 2018), such as heart failure (Madan and Mehra, 2020), hypertension (Yan et al., 2020), and cerebral infarction (Xu et al., 2021). In summary, exposure to BPF can lead to a decrease in the production of SCFAs by the gut microbiota, which may be another mechanism for the induction or aggravation of VC.

Vascular calcification represents a pathological alteration fundamentally driven by chronic inflammation and immune dysregulation (Wu et al., 2013), with its pathogenesis closely linked to the functional interplay between the spleen and liver as pivotal immunomodulatory organs (Emami et al., 2015; Brummer et al., 2025). The unique anatomical connection wherein splenic venous blood directly converges into the liver via the portal vein establishes the physiological foundation of the “spleno-hepatic axis” Research indicates that bisphenol compounds can exploit this axis to trigger systemic chronic inflammatory-immune responses, ultimately impacting the cardiovascular system: First, splenic-derived myeloid-derived suppressor cells (MDSCs) and natural killer T (NKT) cells rapidly infiltrate the liver through portal circulation (Brummer et al., 2025). Simultaneously, damaged hepatocytes release IL-6, GM-CSF, and lipotoxic antigens that retrograde via the portal vein to activate splenic myeloid precursor cells, promoting the expansion and differentiation of MDSCs and NKT cells. This bidirectional pathological cycle leads to the cascade amplification of inflammatory cytokines (TNF-α, IL-1β, IL-6) between the liver and spleen, persistently exacerbating systemic inflammatory processes (Tarantino et al., 2021; Sangwan et al., 2024; Zhu et al., 2024; Brummer et al., 2025). Second, bisphenols synergistically intensify inflammation by disrupting the “gut-spleen axis” Perinatal BPA exposure not only significantly disturbs maternal gut microbiota (e.g., reduced Akkermansia, aberrant Dubosiella) and impairs offspring splenic immune development, but also diminishes the differentiation capacity of splenic regulatory T cells (Tregs) due to decreased microbial metabolite short-chain fatty acids (SCFAs) (López-Moreno et al., 2025). Critically, BPA-induced intestinal barrier damage allows pathogen-associated molecular patterns (PAMPs) like lipopolysaccharide (LPS) to enter the spleen via the portal vein, activating Toll-like receptor 4 (TLR4) and provoking systemic inflammation (López-Moreno et al., 2025). Furthermore, quinone metabolites generated from bisphenol compounds within the spleen deplete antioxidant enzymes (superoxide dismutase, glutathione), inducing lipid peroxidation and DNA damage (Jalal et al., 2018). This subsequently activates the NF-κB pathway and upregulates key inflammatory mediators including cyclooxygenase-2 and inducible nitric oxide synthase, establishing a third independent inflammatory amplification pathway distinct from the aforementioned axes (Jalal et al., 2018). Collectively, bisphenol compounds synergistically drive vascular calcification through three interconnected mechanisms: the spleno-hepatic axis bidirectional pathology, gut-spleen axis disruption, and splenic oxidative stress.

In this study, BPF was selected as the research object based on the ROC curves results. In fact, BPF, BPA, and BPS have similar chemical structures and thus similar physicochemical properties. There is a growing body of evidence that indicates that BPF, BPA and BPS have different degrees of toxicity to organisms. Therefore, we speculate that BPF, BPA and BPS produce the same effect. We look forward to follow-up studies on the roles of BPA and BPS in CVDs. In addition, how BPF causes gut microbiota dysbiosis and how body inflammation triggers VC are worthy of more in-depth study in the future.

## 5 Conclusion

Overall, our results demonstrate that BPF exposure induces mild VC in normal rats and exacerbates VC in VDN-treated rats, mediated through gut microbiota dysbiosis via the gut–vascular axis. This dysbiosis, characterized by elevated lipopolysaccharide (LPS) and reduced short-chain fatty acid (SCFA) levels, triggers inflammatory responses in normal rats and aggravates inflammation in VDN-treated rats. These findings establish a mechanistic link between gut microbiota disruption and BPF-induced VC, providing novel insights into the role of LPS/SCFA imbalance in VC pathogenesis and a theoretical basis for incorporating BPF exposure into cardiovascular disease risk assessments.

## Data Availability

The raw 16S rRNA sequencing data have been deposited in the NCBI Sequence Read Archive (SRA) under BioProject accession PRJNA1287671.

## Footnotes

^1^ http://www.empowerstats.com

^2^ http://www.R-project.org

^3^ www.com.analyses/tools
